# A fast response cataluminescence ether gas sensor based on GO/Mo_2_TiC_2_T_*x*_ at low working temperature

**DOI:** 10.1039/d1ra09356h

**Published:** 2022-03-16

**Authors:** Fakang Pan, Bai Sun, Zhuo Tang, Shuguang Zhu

**Affiliations:** Research Center of Engineering and Technology for Smart City of Anhui Province, School of Environment and Energy Engineering, Anhui Jianzhu University Hefei China panfakang@163.com; Nano-materials and Environmental Detection Laboratory, Hefei Institute of Physical Science, Chinese Academy of Sciences Hefei China bsun@mail.ustc.edu.cn

## Abstract

A cataluminescence (CTL) ether gas sensor based on GO/Mo_2_TiC_2_T_*x*_ composite was developed. The sensor has high selectivity and sensitivity to ether with the response and recovery times of 2 and 8 s, respectively. The optimal operating temperature (155 °C) is low compare with common sensors. Under optimal conditions, the linear range of the concentrations of ether is 9.5–950 ppm; CTL signal intensity and ether concentration show a good linear relationship (*r* = 0.9952); and the detection limit is 0.64 ppm. Furthermore, no response to anything other than acetone after repeatedly tested 10 kinds of common volatile organic compounds, which shows that the sensor has a good selectivity. In addition, the developed sensor has a long life.

## Abstract 2

A cataluminescence (CTL) ether gas sensor based on a GO/Mo_2_TiC_2_T_*x*_ composite was developed. The sensor has high selectivity and sensitivity.

## Graphical abstract

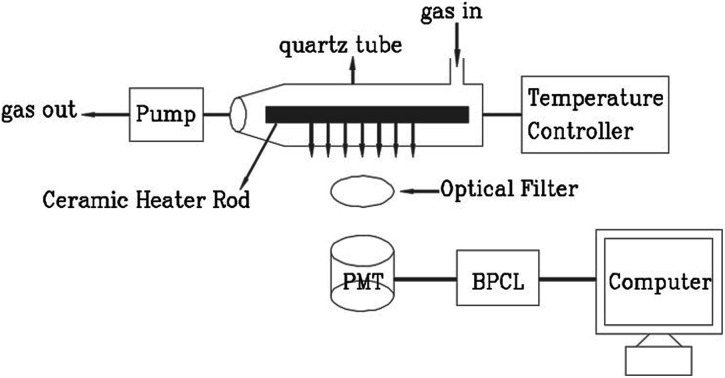

## Introduction

Most of the volatile organic compounds (VOCs) have a pungent smell, which can cause people's sensory discomfort, and do harm to human health and the ecological environment. Ether, as one of the VOCs, can cause fire and explosion accidents. In addition, ether is able to pass through the respiratory tract and be absorbed quickly after entering the alveoli. It can also be absorbed through the skin and quickly enter the brain and adipose tissue through the blood. Long-term exposure to ether is very harmful to the human body. It has harmful effects on the central nervous system, liver and many other organs. Hence, it is necessary to detect ether quickly and accurately. Currently, the most common method for detecting ether is gas chromatography.^1,2^ Although this method has high sensitivity and selectivity, the instrument is large in size, complex in operation and difficult to achieve real-time monitor. Semiconductor metal oxide sensor or surface acoustic wave quartz crystal sensor can also be used to detect ether, which have a good sensitivity but poor selectivity.^3,4^

Cataluminescence (CTL) is the phenomenon of chemiluminescence produced by the catalytic reaction of gas on the surface of solid materials.^5–10^ In 1976, it was first discovered when Breysse *et al.* studied the catalytic oxidation of CO on the surface of ThO_2_, and defined it as “cataluminescence”.^11^ CTL gas sensor has the characteristics of superior selectivity, wide linear range, rapid response and high signal-to-noise ratio, which can monitor gas quickly and accurately. Compared with traditional gas chromatography, it has the characteristics of small size and simple operation. Compared with colorimetry and spectrophotometry, it possesses a continuous monitoring characteristic.

In the 21st century, with the emergence of nanomaterials, the development of CTL has been promoted. Tang *et al.* prepared an acetone highly sensitive and selective sensor based on nano-La_2_O_3_ surface CTL and successfully applied it to the determination of acetone in air samples.^12^ Zhen *et al.* used α-MoO_3_ as a gas-sensitive material to prepare a CTL sensor that can detect ether gas at 120 °C.^13^ Shi *et al.* used aluminum/iron oxide composite to develop a CTL gas sensor for detecting harmful gases such as ether and *n*-hexane, in which the response to ether was observed at 180 °C.^14^ Many nanomaterials-based CTL gas sensors have been reported, such as Sm_2_O_3_, Ag_2_Se, V_2_O_5_, Ti_3_SnLa_2_O_11_.^15–18^

Developing optimal materials with high sensitivity, good selectivity, and mild reaction conditions (at low temperatures) has been an important direction. Two-dimensional materials are beneficial for gas sensing applications, and a larger specific surface area will promote surface reactions. Graphene oxide (GO), as a frequently studied two-dimensional material, has been considered as a potential material for a wide range of applications.^19–21^ Graphene has a stable structure, strong corrosion resistance, and large specific surface area, which is conducive to the composite with other carriers. Two-dimensional transition metal carbides and nitrides, as MXenes, have similar conductivity, adjustable structure and hydrophilicity.^22,23^ The chemical formula of this kind of two-dimensional materials is generally expressed as M_*n*+1_X_*n*_T_*x*_, where M is an early transition metal (*e.g.*, Ti, V, Cr, Nb, Mo, *etc.*), X is carbon or nitrogen, and T_*x*_ represents a terminal functional group, such as –O, –OH or –F.^24^ It was discovered in 2011, and has attracted attention due to its unique physical and chemical properties, but it is currently less used in the field of CTL. Herein, we develop a GO/Mo_2_TiC_2_T_*x*_ composite which is used for gas sensor to detect of ether.^24,25^

## Experimental

### Preparation of experimental materials

All chemicals used in the experiment are of analytical grade and can be used directly without further purification. Concentrated sulfuric acid, graphite powder, sodium nitrate, potassium permanganate, hydrogen peroxide, hydrochloric acid, hydrofluoric acid, absolute ethanol, ether, acetone, carbon tetrachloride, formaldehyde, chloroform, xylene, acetonitrile, ethyl acetate, ammonia and cyclohexane were purchased from Shanghai Group Chemical Reagent Co., Ltd. Mo_2_TiAlC_2_ was purchased from Beijing Beike New Material Technology Co., Ltd.

### Preparation of GO

GO was synthesized by a Hummers' method.^26^ The typical process is as follows: appropriate amount of concentrated sulfuric acid was added in a 250 mL reaction flask in an ice water bath. Under stirring, the solid mixture of 2 g graphite powder and 1 g sodium nitrate were added, and then 6 g potassium permanganate was added. The reaction temperature was controlled below 20 °C, and the solution was stirred. The temperature was increased to about 35 °C and continue stirring for 30 min. Then slowly add a certain amount of deionized water, and raise the temperature to 98 °C. After heating and stirring for 20 min, an appropriate amount of hydrogen peroxide was added to reduce the remaining oxidant and the solution becomes bright yellow. It was washed with 5% HCl solution and deionized water until no sulfate was detected in the filtrate. Finally, the filter cake was fully dried in a vacuum drying oven at 60 °C and stored for further use.

### Preparation of Mo_2_TiC_2_T_*x*_

1.0 g Mo_2_TiAlC_2_ was added to 30 mL hydrofluoric acid (HF) solution. It was stirred continuously for 72 h under the reaction condition of 55 °C. Then the product was washed repeatedly with deionized water, and the centrifuge speed was 4000 rpm, until the pH value of the supernatant was greater than 6. The obtained material was ultrasonically treated for one hour in an argon atmosphere, and filtered and dried to obtain Mo_2_TiC_2_T_*x*_.

### Preparation of GO/Mo_2_TiC_2_T_*x*_

50 mg GO and 50 mg Mo_2_TiC_2_T_*x*_ were dispersed in 50 mL deionized water. The hydrothermal conditions were controlled at 60 °C and stirred continuously for 6 h. Then the material was freeze-dried at −60 °C for 12 h after centrifugation with alcohol and deionized water for several times.

### Characterization

Scanning electron microscopy (SEM) were conducted on a Zeiss Auriga instrument operating at 10 kV. Hitachi H-7650 instrument for transmission electron microscope (TEM) observation at 200 kV, energy dispersive spectrometer (EDS) and X-ray diffraction (XRD, X-Pert powder, Cu Kα) were also used to characterized the samples.

### Apparatus of gas sensor

**Fig. 1:**
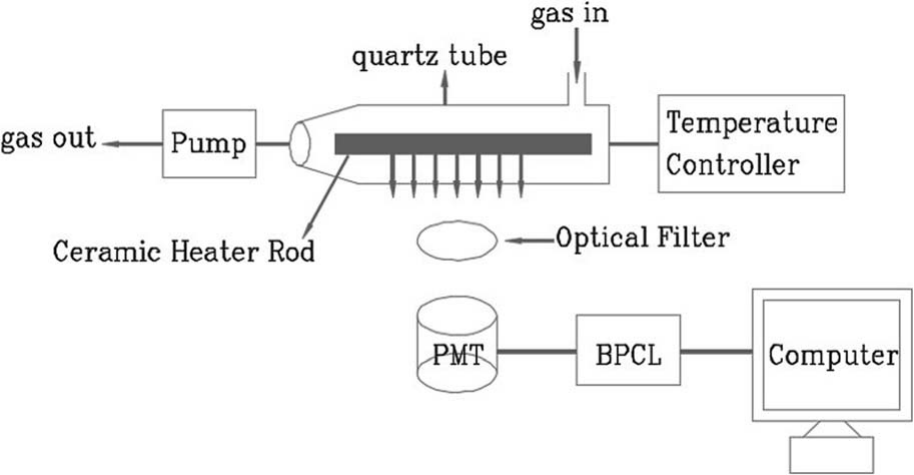
Schematic diagram of the CTL-based sensor system.

Ultra-weak chemiluminescence analyzer (BPCL-1-TIC, Guangzhou Microlight Technology Co., Ltd) was used to detect the CTL signals, the schematic diagram of cataluminescence sensing is shown in the Fig. 1. The whole CTL experimental device is composed of three parts: (1) reaction chamber: it is composed of ceramic heating rod and quartz tube (containing gas inlet and outlet). The gas to be measured flows into the quartz tube from the inlet and fully contacts and reacts with nanomaterials; (2) temperature control system and carrier gas flow rate control system; (3) photoelectric detection and data processing system: BPCL instrument is used to detect, collect and process photoelectric signals, convert weak light signals into electrical signals, and finally transmit the electrical signals to the computer for storage.

## Results and discussions

### Characterization of GO/Mo_2_TiC_2_T_*x*_

**Fig. 2:**
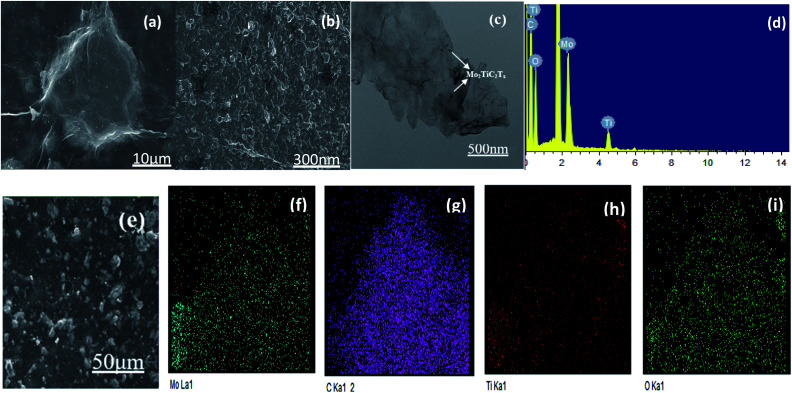
(a) and (b) SEM, (c) TEM images of GO/Mo_2_TiC_2_T_*x*_, (d) EDS spectrum of the GO/Mo_2_TiC_2_T_*x*_, (e)–(i) SEM image and EDS mapping of GO/Mo_2_TiC_2_T_*x*_ catalyst.

**Fig. 3:**
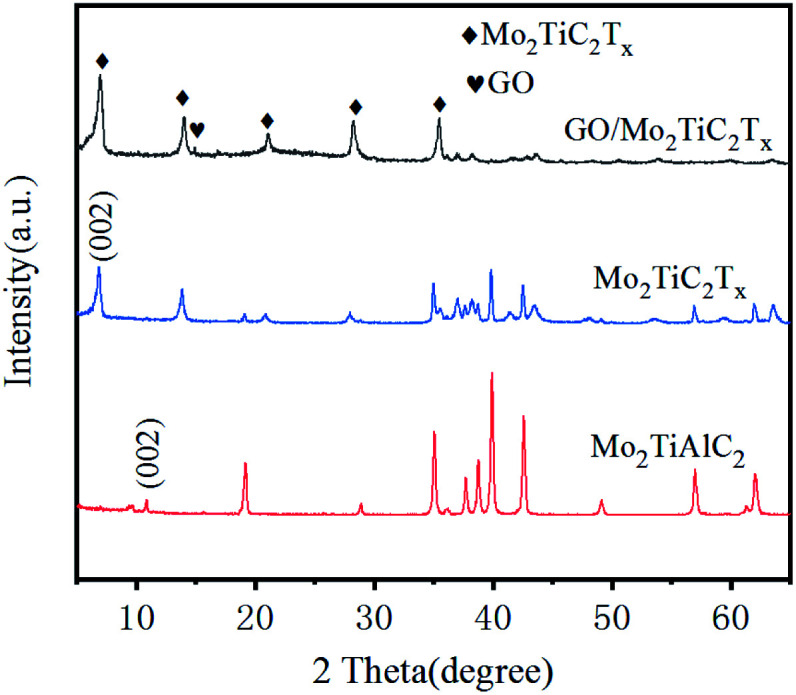
XRD pattern of GO/Mo_2_TiC_2_T_*x*_.

SEM and TEM images of GO/Mo_2_TiC_2_T_*x*_ catalyst are shown in Fig. 2. Fig. 2(a) and (b) show SEM images. It's very clear from the images that the crumpled, monolithic, flaky structure is graphene oxide. It has a large surface area. The Mo_2_TiC_2_T_*x*_ material also presents a sheet-like structure with a small size, forming fish scales shaped that are evenly wrapped on the graphene surface. Fig. 2(b) displays an enlarged view of GO/Mo_2_TiC_2_T_*x*_ composite. Large surface area is conducive to the gas to be tested, and enhances the CTL performance of the sensor. In the EDS spectrum (Fig. 2(d)), we can see that the composite is mainly composed of Ti, O, C, and Mo elements. At the same time, we conducted EDS mapping detection on the material, as shown in Fig. 2(e)–(i). EDS mapping shows the characteristic elements Mo and Ti of Mo_2_TiC_2_T_*x*_, the C and O elements of Mo_2_TiC_2_T_*x*_ and GO. It can be observed that Mo and Ti are uniformly distributed, indicating that Mo_2_TiC_2_T_*x*_ is uniformly attached to the GO. The (002) peak of Mo_2_TiC_2_T_*x*_ powders left shifts to 6.9° from 10.9° of Mo_2_TiAlC_2_T_*x*_ as shown in XRD patterns (Fig. 3), indicating the larger *d*-spacing of Mo_2_TiC_2_T_*x*_ than that of Mo_2_TiAlC_2_T_*x*_ because of introduced groups during the etching process. The surface of MXene prepared by solution etching is generally accompanied by functional groups. T represents a terminal functional group, such as –O, –OH or –F, which indicate the T of Mo_2_TiC_2_T_*x*_ in our study would be –O, –OH or –F.^24^ The characteristic peak of GO was observed at 2*θ* = 15°. The elemental composition of GO/Mo_2_TiC_2_T_*x*_ are listed in Table 1.

**Table 1:** Elemental composition of the synthesized GO/Mo_2_TiC_2_T_*x*_ nanoparticle

Elements	Weight%	Atomic%
CK	4.19	69.58
OK	2.08	25.92
TiK	0.32	1.33
Mo	1.52	3.17
Totals	8.11	100

### Sensing performance towards ether

**Fig. 4:**
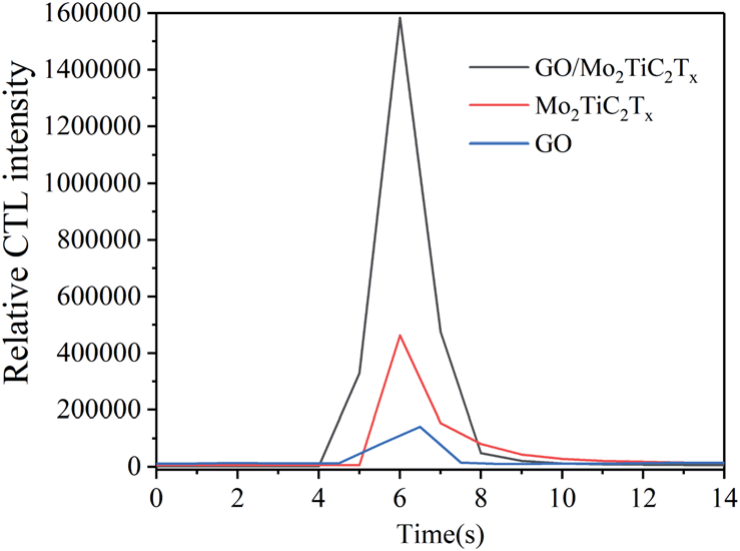
Comparison of CTL performance of different materials (concentration: 950 ppm; flow rate: 250 mL min^−1^; temperature: 165 °C).

The catalytic luminescence properties of GO, Mo_2_TiC_2_T_*x*_ and GO/Mo_2_TiC_2_T_*x*_ were measured, respectively. The results are shown in Fig. 4. Compared with the Mo_2_TiC_2_T_*x*_, the hybrid structure shows significantly enhanced sensing performance. It indicates that the addition of GO can effectively enhance the signal intensity of GO/Mo_2_TiC_2_T_*x*_-based sensor for detecting aether. The improved sensing performance is attributed to the effective combination of GO and Mo_2_TiC_2_T_*x*_. In our study, the GO/Mo_2_TiC_2_T_*x*_ composite was investigated as follows.

**Fig. 5:**
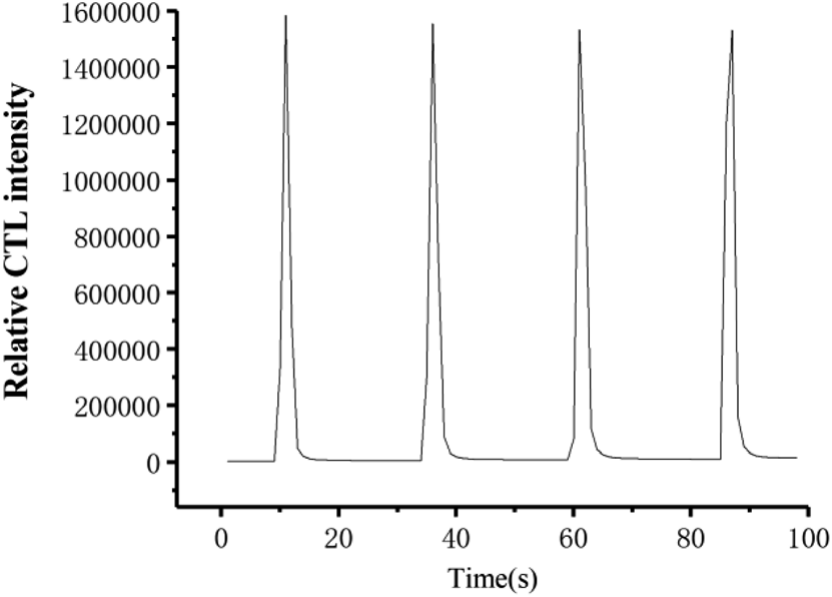
Typical CTL spectra at optimum conditions. (concentration: 950 ppm; flow rate: 400 mL min^−1^; the temperature: 155 °C.)

Fig. 5 shows the response curve towards ether on the sensor surface under optimal conditions. The response and recovery times are within 2 and 8 s, respectively; and the signal intensity is high. It can be seen that the sensor has the advantages of high sensitivity and fast response to ether. In Fig. 5, the relative standard deviation (R.S.D.) of the intensity of 950 ppm ether vapor measured four times is 2.1%. The results show that the CTL signal intensity of ether on the material surface is stable, and the reproducibility is good. The excellent sensing performance can be attributed to its two-dimensional structure and large surface area, which is favourable to transfer of electrons, and improve adsorption and desorption of O_2_.

### Effect of working temperature

**Fig. 6:**
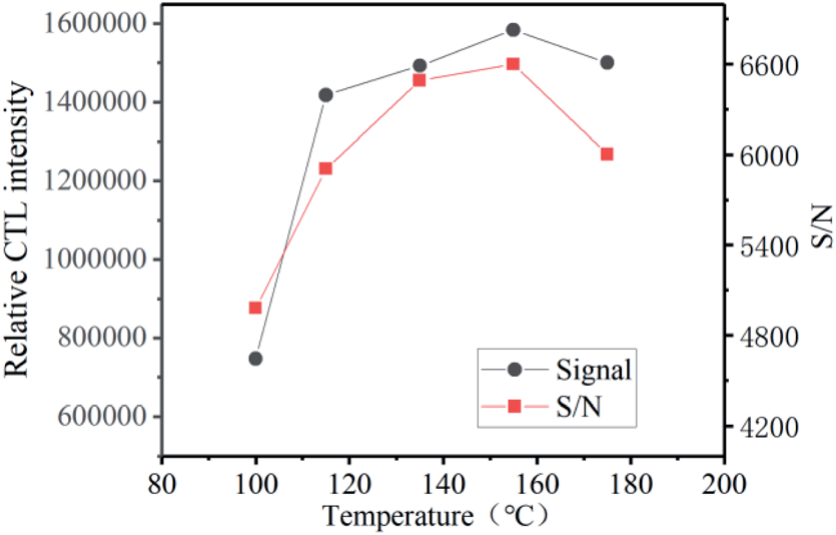
Effect of working temperature on CTL intensity, and S/N.

Temperature plays an important role in the CTL process. Because the catalyst has a lower catalytic activity at lower temperature, external conditions are required to increase the temperature. As shown in Fig. 6, in order to exert the best performance of the material, the relationship between the CTL intensity of ether on the surface of the composite material and the temperature under the optimal conditions of flow rate and concentration. In the range from 100 °C to 155 °C, the CTL signal strength increases with the increase of temperature. The signal value reaches the maximum at 155 °C, and then the temperature rises, which has little effect on the signal strength, the intensity drops slightly. The noise is also not conducive to our detection. We can observe that, with the increase of temperature, not only the CTL signal strength increases, but also the noise value increases, which causes the S/N to increase first and then decrease with the increasing of temperature. The signal-to-noise ratio (S/N) reaches its maximum at 155 °C, which indicates it is the best working temperature.

We can see from the Fig. 6 that ether reacts at a relatively lower temperature, as low as 100 °C. Low-temperature CTL has always been a thorny problem in this field. At present, many CTL sensors need to work at high temperatures, and researchers have been exploring low-temperature materials. Compared with previous reports, the sensor based on GO/Mo_2_TiC_2_T_*x*_ has a low working temperature and high signal intensity (Table 2).^12,14–18^

**Table 2:** Optimum temperature for different catalysts

Catalysts	Analytes	Optimum temperature (°C)	References
Nano-La_2_O_3_	Acetone	361	12
γ-Al_2_O_3_/Fe(OH)_3_	Ether	180	14
Sm_2_O_3_	Isobutyraldehyde	177	15
Ag_2_Se	Carbon tetrachloride	240	16
V_2_O_5_	*tert*-Butyl mercaptan	351	16 and 17
Ti_3_SnLa_2_O_11_	Formaldehyde and ammonia	350	18
GO/Mo_2_TiC_2_T_*x*_	Ether	155	This study

### Effect of air flow

**Fig. 7:**
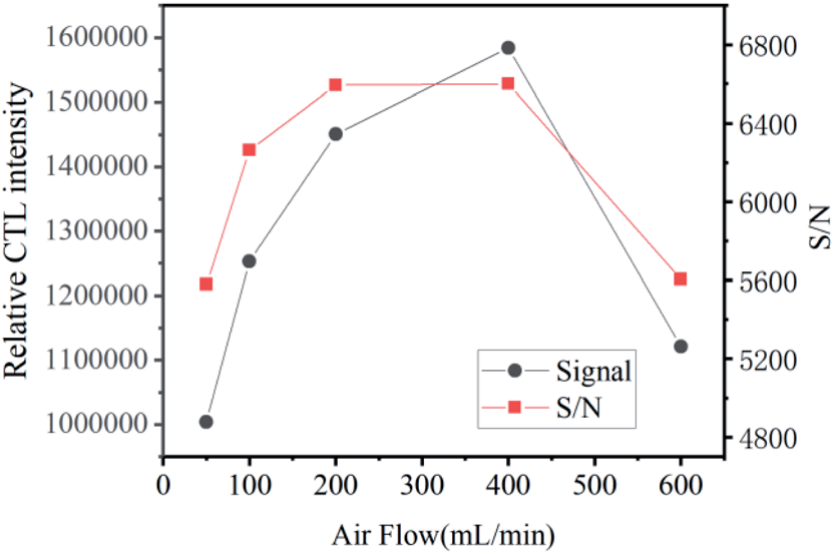
Effect of working temperature on CTL intensity, and S/N.

The flow rate of carrier gas also has an effect on the CTL intensity. The relationship between carrier gas velocity and CTL strength was investigated when the temperature was 155 °C and the concentration of ether gas was certain, in the flow rate range of 50–600 mL min^−1^. It can be seen from Fig. 7 that the CTL response has the highest intensity when the carrier gas flow rate is 400 mL min^−1^. When the flow rate is less than 400 mL min^−1^, the carrier gas flow rate increases, and the ether gas in contact with the material surface per unit time increases with the increase of the flow rate. Therefore, the CTL signal shows an upward trend, indicating that the reaction rate is limited by the rate when the ether gas transfers to the catalyst surface. With the further increase of the flow rate, when it exceeds 400 mL min^−1^, part of the ether gas has been taken away from the reaction chamber before it contacts the surface of the material, resulting in a low signal. Combined with the value of signal-to-noise ratio and comprehensive consideration, it is considered that 400 mL min^−1^ would be the best flow rate.

### Analysis characteristic

**Fig. 8:**
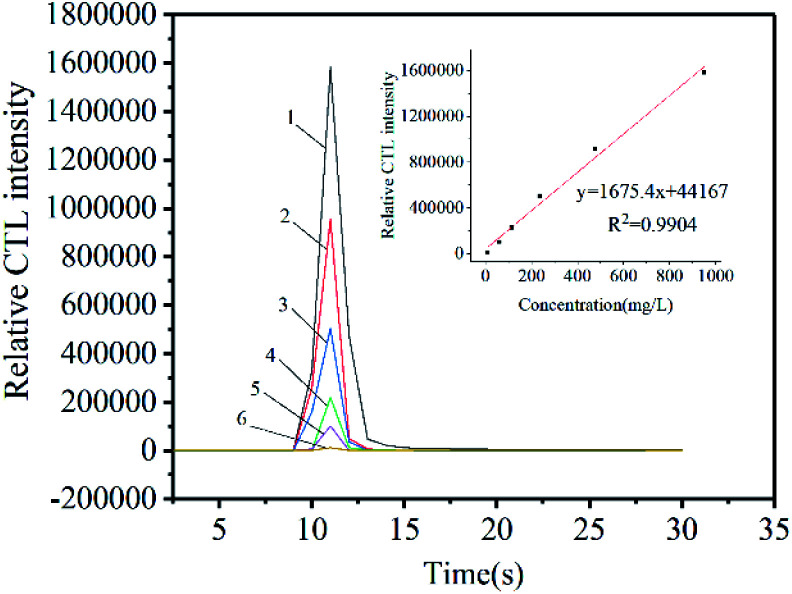
CTL spectra and calibration curve between CTL intensity and ether concentration. (1 : 950 ppm, 2 : 475 ppm, 3 : 235 ppm, 4 : 115 ppm, 5 : 60 ppm, 6 : 9.5 ppm.)

In order to further study the CTL properties under the optimal conditions, different concentrations of ether were injected into the reaction chamber. The linear relationship between CTL strength and the concentration of ether was established, as shown in Fig. 8. In the concentration range of 9.5–950 ppm, we can observe that the CTL intensity is proportional to the concentration of ether. The linear equation is *y* = 1675.4*x* + 44 167 (*R*^2^ = 0.9904, *n* = 6), where *x* represents the concentration of ether, *y* represents the intensity of CTL signal, and *R* is the regression coefficient. The limit of detection (LOD) to ether is 0.64 ppm (S/N = 3). The GO/Mo_2_TiC_2_T_*x*_ composite developed here has a lower LOD for ether, which is lower than some other sensors, such as ZnWO_4_, SiO_2_/Fe_3_O_4_, α-MoO_3_, Mg–Al LDO^5,6,13,27,28^ (Table 3).

**Table 3:** Detection limits of different catalysts for ether

Analytes	Catalysts	Linear range	LOD	References
Ether	ZnWO_4_	20–3500 ppm	8.7 ppm	5
	SiO_2_/Fe_3_O_4_	10–3000 ppm	6.7 ppm	6
	α-MoO_3_	9.0–2000 ppm	7.5 ppm	13
	Mg–Al LDO	0.1–8.0 mM	0.02 mM	27 and 28
	GO/Mo_2_TiC_2_T_*x*_	9.5–950 ppm	0.64 ppm	This study

### Selectivity and life of materials

**Fig. 9:**
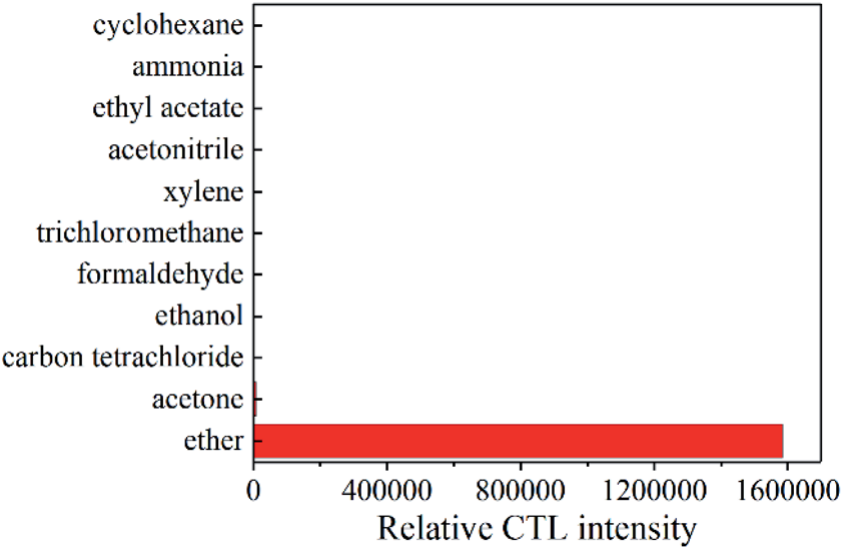
CTL response of sensor to different compounds.

In order to study the selectivity of the GO/Mo_2_TiC_2_T_*x*_ composites. Under optimal conditions, we tested 10 common volatile organic compounds that may coexist with ether, including acetone, carbon tetrachloride, ethanol, formaldehyde, chloroform, xylene, acetonitrile, ethyl acetate, ammonia, and cyclohexane. In Fig. 9, none of these gases react on the surface of the material except acetone. Although acetone does react, the signal strength is negligible compared to ether, because the interference of acetone is less than 1%. It indicates a high selectivity to ether.

Within 30 days, the sensor was tested once a week. Under the best conditions, 950 ppm of ether was injected into the reaction chamber. By analysing each test, it is found that the GO/Mo_2_TiC_2_T_*x*_ composite hardly changes the CTL signal intensity towards ether. The results shows that the gas sensor has a good stability for long-term use.

### Possible sensing mechanism

At present, there are few studies on the mechanism of CTL, mainly due to the complex reaction process of CTL reaction. Thorough research on the mechanism of CTL is of great significance for the controllable synthesis of catalysts with high sensitivity and strong selectivity. A CTL mechanism that has been accepted by many researchers, which is the formation of highly active endoperoxides during the reaction.^29–39^ It is considered that GO/Mo_2_TiC_2_T_*x*_ has sensitive properties to aether because of the reaction of aether with the catalyst to form highly reactive intermediates. Although other gases can also be catalyzed, the amount of CTL intermediates generated by ether is much greater than others, resulting in a good sensitivity to ether. According to Zhang's report, ether is able to be oxidized to excite the species acetaldehyde and CO_2_ molecules.^40^ The possible CTL mechanism of ether is as follows: under the catalyst conditions, O_2_ is adsorbed on the surface of GO/Mo_2_TiC_2_T_*x*_ composite, which captures electrons to generate negative oxygen species. Then, it reacts with the ether gas to produce an excited state CH_3_CHO molecule, and reacts with oxygen to generate an excited state CO_2_, finally both of them return to the ground state and generate a luminescent signal. The reactions involved are presented as follows:^41–43^O_2_ + e^−^ → O_2_^−^C_2_H_5_OC_2_H_5_ + O_2_^−^ → CH_3_CHO* + O_2_ → CH_3_CHO + *hν*
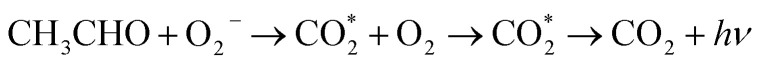


## Conclusions

A new CTL sensing material is developed by combining two-dimensional graphene oxide and Mo_2_TiC_2_T_*x*_. The ether sensor based on the composite can not only work at a low temperature, but also has high sensitivity and selectivity with the fast response during detection. The results show that GO/Mo_2_TiC_2_T_*x*_ is a potential candidate with low operating cost and good stability, which can be applied to the real detection towards ether gas.

## Author contributions

Fakang Pan: conceptualization, methodology, data curation, investigation, writing – review & editing. Bai Sun: methodology, data curation, investigation, project administration, supervision. Zhuo Tang: data curation, formal analysis, investigation, writing – original draft. Shuguang Zhu: conceptualization, methodology, supervision.

## Conflicts of interest

There are no conflicts to declare.

## Supplementary Material
